# Supplemental Nutrition Assistance Program Access and Racial Disparities in Food Insecurity

**DOI:** 10.1001/jamanetworkopen.2023.20196

**Published:** 2023-06-26

**Authors:** Laura J. Samuel, Deidra C. Crews, Bonnielin K. Swenor, Jiafeng Zhu, Elizabeth A. Stuart, Sarah L. Szanton, Boeun Kim, Pallavi Dwivedi, Qiwei Li, Nicholas S. Reed, Roland J. Thorpe

**Affiliations:** 1 Johns Hopkins School of Nursing, Baltimore, Maryland; 2 Division of Nephrology, Johns Hopkins School of Medicine, Baltimore, Maryland; 3 The Johns Hopkins Disability Health Research Center, Baltimore, Maryland; 4 Bloomberg School of Public Health, Johns Hopkins University, Baltimore, Maryland; 5 Johns Hopkins School of Medicine, Baltimore, Maryland; 6 College of Health and Human Services, University of California, Fresno

## Abstract

**Importance:**

Racially minoritized people experience disproportionately high rates of food insecurity. The Supplemental Nutrition Assistance Program (SNAP) reduces food insecurity.

**Objective:**

To evaluate SNAP access with regard to racial disparities in food insecurity.

**Design, Setting, and Participants:**

This cross-sectional study used data from the 2018 Survey of Income and Program Participation (SIPP). On the basis of random sampling strategies, 44 870 households were eligible for the SIPP, and 26 215 (58.4%) participated. Sampling weights accounted for survey design and nonresponse. Data were analyzed from February 25 to December 12, 2022.

**Exposures:**

This study examined disparities based on household racial composition (entirely Asian, entirely Black, entirely White, and multiple races or multirace based on SIPP categories).

**Main Outcomes and Measures:**

Food insecurity during the prior year was measured using the validated 6-item US Department of Agriculture Food Security Survey Module. SNAP participation during the prior year was classified based on whether anyone in the household received SNAP benefits. Modified Poisson regression tested hypothesized disparities in food insecurity.

**Results:**

A total of 4974 households that were eligible for SNAP (income ≤130% of the poverty threshold) were included in this study. A total of 218 households (5%) were entirely Asian, 1014 (22%) were entirely Black, 3313 (65%) were entirely White, and 429 (8%) were multiracial or of other racial groups. Adjusting for household characteristics, households that were entirely Black (prevalence rate [PR], 1.18; 95% CI, 1.04-1.33) or multiracial (PR, 1.25; 95% CI, 1.06-1.46) were more likely to be food insecure than entirely White households, but associations differed depending on SNAP participation. Among households that did not participate in SNAP, those that were entirely Black (PR, 1.52; 97.5% CI, 1.20-1.93) or multiracial (PR, 1.42; 97.5% CI, 1.04-1.94) were more likely to be food insecure than White households; however, among SNAP participants, Black households were less likely than White households to be food insecure (PR, 0.84; 97.5% CI, 0.71-0.99).

**Conclusions and Relevance:**

In this cross-sectional study, racial disparities in food insecurity were found among low-income households that do not participate in SNAP but not among those that do, suggesting that access to SNAP should be improved. These results also highlight the need to examine the structural and systemic racism in food systems and in access to food assistance that may contribute to disparities.

## Key Points

**Question:**

Does the Supplemental Nutrition Assistance Program (SNAP) address racial disparities in food insecurity?

**Findings:**

In this cross-sectional study of 4974 US households, Black and multiracial households had higher rates of food insecurity than White households in adjusted analyses. This disparity was not found among households that had access to SNAP benefits.

**Meaning:**

These findings suggest that SNAP likely plays a key role in addressing food insecurity, but there are racial disparities in food insecurity among those not participating in the program.

## Teaser

This cross-sectional study examines racial disparities in food insecurity overall and compared households participating in the Supplemental Nutrition Assistance Program with those not participating.

## Introduction

Black individuals have had consistently higher food insecurity rates during the past 20 years than White individuals in the US, even after accounting for relatively lower mean income levels.^1,2^ However, national data examining racial disparities in food insecurity have not accounted for Supplemental Nutrition Assistance Program (SNAP) participation. This is an important gap for 2 reasons. First, longitudinal studies^3,4,5^ applying quasi-experimental methods have shown that SNAP participation reduces food insecurity by providing money for food, and there is also good evidence that households that are food insecure are more likely to enroll in SNAP than food-secure households.^4^ Therefore, SNAP may play a key role in attenuating racial disparities in food insecurity by addressing economic inequality, but these potential effects have not been considered in prior studies. Second, there is conflicting information about racial disparities in SNAP access. As examples, 1 study found that parents of children who are Black were less likely to be consistently enrolled in SNAP than parents of children who were White,^6^ 1 study documented lower SNAP participation rates in predominately Black Southern states,^7^ and 1 study in the Baltimore City area found that individuals who are Black were more likely to participate in SNAP than those who are White.^8^ Overall, there is a lack of attention paid in national data to the role of SNAP in addressing racial disparities in food insecurity.

There is good rationale to evaluate the role of SNAP because of evidence of racially segregated access to healthy foods.^9,10,11,12,13^ There is substantial geographic variation in the price of basic healthy food items^14^ and some evidence that predominately Black neighborhoods have higher prices for items such as low-fat milk and fruits and vegetables.^15^ Although there has been an increasing recognition of the ways that structural racism in the health care sector has shaped health inequities^16^ and an increasing recognition that food insecurity is a public health issue,^17,18^ there is a lack of attention to the ways that systemic racism in food systems and food assistance programs may shape food insecurity inequities. Therefore, this study examined the interrelationships among racial background, SNAP access, and food insecurity among households that are income eligible for the SNAP program (incomes ≤130% of the federal poverty threshold). Specifically, this study examined racial disparities in food insecurity overall and compared households participating in SNAP with those not participating.

## Methods

### Study Design and Sample

The Survey of Income and Program Participation (SIPP) provides nationally representative, household-level US data and is described in detail elsewhere.^19^ Briefly, SIPP uses multistage stratified sampling to recruit a sample that is representative of the civilian, noninstitutionalized US population. This cross-sectional study used data from the 2018 SIPP, which is the most recent year of complete national data before the COVID-19 pandemic. Of the 44 870 eligible households, 26 215 (58.4%) provided written informed consent and participated in interviews. The unit of analysis is households rather than individuals because food insecurity and SNAP access are both measured at the household level. This study included the 4974 households with incomes at 130% or lower of the federal poverty threshold for 3 reasons. First, everyone below this threshold is eligible for SNAP based on federal guidelines. Second, individuals with incomes below this threshold have rates of food insecurity that are 3 times higher than the general population.^20^ Third, although some states allow households with higher incomes to participate in SNAP, only approximately 6% of SNAP participants have incomes over this threshold.^21^ There were no missing data for these analyses. The Johns Hopkins Medicine institutional review board determined this study was exempt from review because it was not human participant research. This study followed the Strengthening the Reporting of Observational Studies in Epidemiology (STROBE) reporting guideline.

### Key Measures

Key household variables for this study are food insecurity, SNAP access, and composition of racial background (see the eTable in Supplement 1 for survey questions). Household food insecurity status during the past year was classified using the validated 6-item US Department of Agriculture Food Security Survey Module.^22^ SNAP access variables included SNAP participation during the past year and, among participants, benefit duration and amount. SNAP participation was classified based on whether anyone in the household reported that the household received SNAP benefits during the past 12 months. Among SNAP-participating households, benefit duration was measured as the number of months of benefits received during the past year. Per-person benefit amount was calculated by dividing the household benefit amount by the total number of people in the household during the month. The household composition of racial backgrounds was categorized as entirely Asian, entirely Black, entirely White (reference), and multiple races or multirace individuals, which included other racial groups (American Indian, Native Hawaiian, and Pacific Islander) in the household based on SIPP categories.

### Additional Household Characteristics

Variables that may be associated with racial background and either food insecurity or SNAP participation were used in this study. These variables included the number of adults in the household (≤1 [reference], 2, or ≥3), US region (Northeast, Midwest [reference], South, and West), nativity status (all born in the US [reference], all born outside the US, or a mixture), and ethnicity (entirely Hispanic, entirely non-Hispanic [reference], or both). Because household SNAP eligibility depends partly on the presence of children younger than 18 years or adults 60 years or older, household indicators (yes or no [reference]) for the presence of children and the presence of older adults were both reported. Although eligibility can also depend on disability status, disability was not considered in these analyses because it likely lies on the relevant causal pathways attributable to racial disparities in disability prevalence.^23^ Because SNAP benefit amounts depend partly on household income, we also used the reported total monthly household income, which includes the sum of earnings and other sources of income (ie, Temporary Assistance for Needy Families, Supplemental Security Income, and unemployment).

### Statistical Analysis

Data were analyzed from February 25 to December 12, 2022. The probability of both food insecurity and SNAP participation were estimated for each racial group. Poisson regression models with robust SEs, which are recommended for commonly occurring binary outcomes,^24^ were used to estimate and compare the prevalence of food insecurity by racial groups for the primary analyses. Secondary analyses evaluated whether SNAP is associated with racial disparities in food insecurity using Poisson regression models stratified by SNAP participation. Models were adjusted for all additional household characteristics. Additional analyses compared SNAP duration and benefit amounts based on racial groups among households that received SNAP in the past year and the past month using the Kruskal-Wallis test. Household-level sampling weights were applied to all analyses so that inferences could be drawn to all US households with incomes at 130% or less of the federal poverty level, and variance estimates account for the complex survey design. Analyses were conducted in R, version 4.2.2 (R Foundation for Statistical Computing) using a 2-sided α = .05, except that SNAP subgroup analyses corrected for 2 tests (α = .025).

## Results

A total of 4974 households that were eligible for SNAP (income ≤130% of the poverty threshold) were included in this study. A total of 218 households (5%) identified entirely as Asian, 1014 (22%) as entirely Black, 3313 (65%) as entirely White, and 429 (8%) as multiracial or other racial groups (Table 1). Although households that were entirely Black were 46% more likely to participate in SNAP (prevalence rate [PR], 54%) and multiracial households were 30% more likely to participate in SNAP (PR, 48%) than entirely White households (PR, 37%) (*P* < .001), food insecurity rates were also 20% higher in Black households (PR, 30%) and 28% higher in multiracial households (PR, 32%) than in entirely White households (PR, 25%) (*P* < .001) (Figure). Households that were entirely Black were also more likely to be in the South (58%) (*P* < .001) than households that were entirely White (39%) or Asian (22%), or households that were multiracial (35%). Households that were either entirely Black (7%) or multiracial (3%) were less likely to be composed of individuals born outside the US than households that were entirely White (9%) or Asian (62%) (*P* < .001).

**Table 1.** Characteristics of Households Overall and Based on the Composition of Racial Background Among Households Likely Eligible for Supplemental Nutrition Assistance Program (Incomes ≤130% of the Poverty Threshold) Participating in the 2018 Survey of Income and Program Participation^a^

Characteristic	Overall (N = 4974)	Entirely White (n = 3313) (65%)	Entirely Black (n = 1014) (22%)	Entirely Asian (n = 218) (5%)	Other racial groups and multiracial (n = 429) (8%)^b^	*P* value^c^
Ethnicity						
Entirely Hispanic	931 (18.1)	821 (24.0)	54 (5.8)	5 (2.2)	51 (12.0)	<.001
Entirely non-Hispanic	3860 (78.1)	2387 (72.7)	938 (91.8)	208 (95.1)	327 (75.7)	
Both	183 (3.9)	105 (3.3)	22 (2.4)	5 (2.8)	51 (12.3)	
US region						
Northeast	731 (17.3)	484 (17.7)	143 (15.0)	46 (24.7)	58 (15.7)	<.001
Midwest	975 (21.4)	669 (21.7)	186 (21.9)	30 (13.9)	90 (21.9)	
South	2218 (41.5)	1393 (38.4)	616 (57.7)	48 (22.0)	161 (35.2)	
West	1050 (19.8)	767 (22.2)	69 (5.4)	94 (39.4)	120 (27.4)	
No. of adults in household						
≤1	2917 (58.1)	1856 (55.2)	759 (74.6)	99 (44.1)	203 (46.2)	<.001
2	1593 (32.4)	1141 (345.0)	193 (19.3)	88 (41.6)	171 (41.1)	
≥3	464 (9.5)	316 (9.8)	62 (6.1)	31 (14.3)	55 (12.8)	
Children in the household						
No	3158 (62.2)	2151 (64.3)	630 (58.8)	157 (72.1)	220 (49.6)	<.001
Yes	1816 (37.8)	1162 (35.7)	384 (41.2)	61 (27.9)	209 (50.4)	
Household members born in the US						
All born outside US	504 (10.5)	290 (9.0)	62 (6.9)	140 (62.3)	12 (2.8)	<.001
All born in US	3861 (77.3)	2578 (77.8)	902 (87.7)	28 (14.1)	353 (80.8)	
Both	609 (12.3)	445 (13.2)	50 (5.5)	50 (23.7)	64 (16.4)	
Anyone in the household aged ≥60 y						
No	3112 (66.4)	2040 (64.8)	638 (68.8)	138 (66.8)	296 (72.9)	.002
Yes	1862 (33.6)	1273 (35.2)	376 (31.2)	80 (33.2)	133 (27.1)	
Monthly household income, mean (SE), US$	953 (27)	981 (34)	776 (61)	705 (92)	1314 (86)	<.001

^a^ Data are presented as number (percentage) of households unless otherwise indicated. Household sampling weights were applied to percentage estimates and statistical tests but not to frequency counts so that inferences can be drawn to US households in 2018, and variance estimates account for the complex survey design.

^b^ Other racial groups include American Indian, Native Hawaiian, and Pacific Islander.

^c^ The χ^2^ tests were used to generate all *P* values except for household income, which used the Kruskal-Wallis test.

**Figure.**
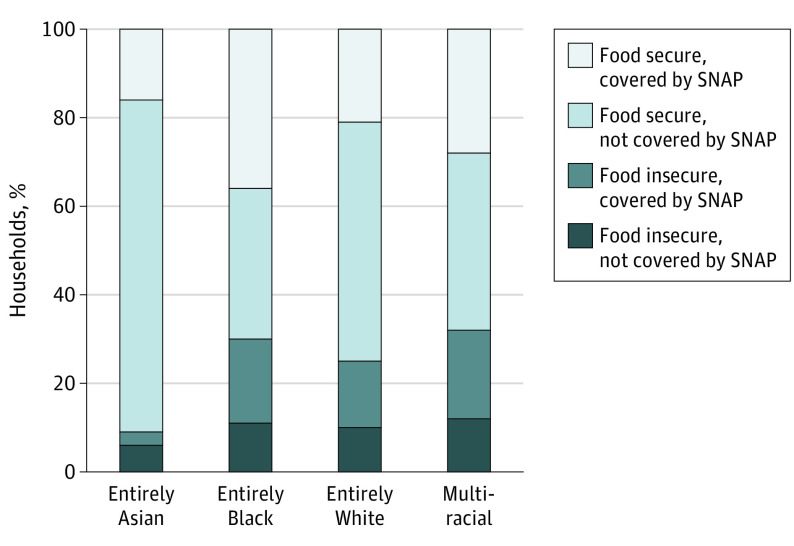
Food Insecurity and Supplemental Nutrition Assistance Program (SNAP) Participation by Racial Identity Estimated combined probability of food insecurity and SNAP participation based on the composition of racial identity among households likely eligible for SNAP (incomes ≤130% of the poverty threshold) participating in the 2018 Survey of Income and Program Participation. The combined probabilities of SNAP participation and food insecurity differ across racial groups. Household sampling weights were applied so that inferences can be drawn to US households with incomes at 130% or less of the poverty threshold in 2018 and variance estimates account for the complex survey design.

In this sample of households at or near the poverty limit (≤130% of the poverty threshold), mean monthly incomes were $333 lower in entirely White households, $538 lower in entirely Black households, and $609 lower in entirely Asian households than multiracial households (*P* < .001), which had the highest monthly income levels ($1314). However, multiracial households were also typically larger; they were the most likely to have children (51%; *P* < .001) and were among the least likely to have only 1 adult (46%; *P* < .001) compared with those households that were entirely Black (42% vs 75%), entirely White (36% vs 55%), or entirely Asian (28% vs 44%). Among SNAP-participating households, there were no racial differences in the duration of SNAP benefits or per-person benefit amounts (Table 2).

**Table 2.** SNAP Access Characteristics, Overall and Based on the Composition of Racial Background Among SNAP-Participating Households in the 2018 Survey of Income and Program Participation^a^

Characteristics	Mean (SE)					*P* value
	Overall (N = 2052)	All White (n = 1252)	All Black (n = 554)	All Asian (n = 40)	Other racial groups or multiracial (n = 206)^b^	
Duration of SNAP benefits among households that received SNAP in the past year, mo	11.00 (0.1)	10.98 (0.1)	10.97 (0.1)	11.28 (0.4)	11.14 (0.2)	.76
Per-person SNAP benefit amount among households that received SNAP during the survey month, US$	106.4 (1.3)	105.62 (2.2)	110.16 (2.5)	110.66 (9.1)	99.16 (3.8)	.08

Abbreviation: SNAP, Supplemental Nutrition Assistance Program.

^a^ Household sampling weights were applied so that inferences can be drawn to US households in 2018 and variance estimates account for the complex survey design. The Kruskal-Wallis test was used to estimate differences across groups.

^b^ Other racial groups include American Indian, Native Hawaiian, and Pacific Islander.

When stratifying based on household racial composition, the combined rates of food insecurity and SNAP participation varied based on racial background (Figure). As examples, rates of being food insecure and not participating in SNAP were higher among households that were entirely Black (11%) or multiracial (12%) than those that were entirely White (10%) (*P* < .001). Similarly, rates of being food insecure while participating in SNAP were 27% higher among households that were entirely Black (PR, 19%) and 33% higher among multiracial households (PR, 20%) than entirely White households (PR, 15%; *P* < .001).

Poisson regression models adjusting for additional household characteristics also found evidence of racial disparities but only among those not participating in SNAP. Among all households, those that were entirely Black had an 18% higher risk of being food insecure (PR, 1.18; 95% CI, 1.04-1.33), and multiracial households had a 25% higher risk (PR, 1.25; 95% CI, 1.06-1.46) than households that were entirely White (Table 3). Among households not participating in SNAP, those that were entirely Black had a 52% higher risk of food insecurity (PR, 1.52; 97.5% CI, 1.20-1.93), and multiracial households had a 42% higher risk (PR, 1.42; 97.5% CI, 1.04-1.94) than entirely White households. However, among SNAP participating households, multiracial households no longer had a higher risk of food insecurity when compared with White households (PR, 1.03; 97.5% CI, 0.83-1.27), and those that were entirely Black had a 16% lower rate (PR, 0.82; 97.5% CI, 0.71-0.99).

**Table 3.** Adjusted Associations Between Racial Background With Food Insecurity Among Households Likely Eligible for SNAP (Incomes ≤130% of the Poverty Threshold) Participating in the 2018 Survey of Income and Program Participation^a^

Characteristic	Total sample PR (95% CI) (N = 4973)	SNAP participating (n = 2052), PR (97.5% CI)^b^	Non–SNAP participating (n = 2922), PR (97.5% CI)^b^
Race			
Entirely White	1 [Reference]	1 [Reference]	1 [Reference]
Entirely Black	1.18 (1.04-1.33)	0.84 (0.71-0.99)	1.52 (1.20-1.93)
Entirely Asian	0.48 (0.31-0.75)	0.49 (0.22-1.07)	0.60 (0.31-1.16)
Other racial groups or multiracial^c^	1.25 (1.06-1.46)	1.03 (0.83-1.27)	1.42 (1.04-1.94)
Ethnicity			
Entirely Hispanic	1 [Reference]	1 [Reference]	1 [Reference]
Entirely non-Hispanic	0.87 (0.74-1.01)	1.05 (0.85-1.29)	0.84 (0.62-1.13)
Both	1.21 (0.95-1.55)	1.18 (0.83-1.68)	1.46 (0.94-2.28)
US region			
Midwest	1 [Reference]	1 [Reference]	1 [Reference]
Northeast	0.92 (0.78-1.10)	0.95 (0.76-1.19)	0.73 (0.50-1.06)
South	1.03 (0.90-1.17)	1.09 (0.92-1.30)	1.00 (0.78-1.29)
West	0.89 (0.76-1.05)	1.02 (0.81-1.27)	0.90 (0.67-1.21)
No. of adults in the household			
≤1	1 [Reference]	1 [Reference]	1 [Reference]
2	0.87 (0.77-0.98)	0.96 (0.82-1.12)	0.79 (0.62-0.99)
≥3	0.93 (0.77-1.12)	1.14 (0.90-1.45)	0.82 (0.58-1.16)
Children in the household			
No	1 [Reference]	1 [Reference]	1 [Reference]
Yes	1.05 (0.93-1.18)	0.76 (0.64-0.89)	1.21 (0.96-1.52)
Household nativity			
All born outside US	1 [Reference]	1 [Reference]	1 [Reference]
All born in US	0.73 (0.58-0.92)	0.73 (0.51-1.05)	0.86 (0.59-1.24)
Both	0.71 (0.58-0.87)	0.87 (0.66-1.14)	0.66 (0.46-0.95)
Anyone in the household aged ≥60 y			
No	1 [Reference]	1 [Reference]	1 [Reference]
Yes	0.77 (0.69-0.87)	0.75 (0.64-0.88)	0.72 (0.57-0.90)
Monthly household income (in thousands)	1.02 (1.00-1.05)	1.01 (0.95-1.07)	1.02 (0.99-1.06)

Abbreviations: PR, prevalence rate; SNAP, Supplemental Nutrition Assistance Program.

^a^ Estimates obtained from Poisson regression model with robust standard errors. Household food insecurity was measured using the 6-item USDA Food Security Survey Module.^22^ Models adjusted for all independent variables listed in the table. Household sampling weights were applied so that inferences can be drawn to US households in 2018 and variance estimates account for the complex survey design.

^b^ To correct for multiple tests, 97.5% CIs were used based on α/2 = .025.

^c^ Other racial groups include American Indian, Native Hawaiian, and Pacific Islander.

## Discussion

This cross-sectional study builds on prior research^1,2^ documenting racial disparities in food insecurity by showing that racial disparities differ depending on SNAP participation among low-income US households. Specifically, racial disparities in food insecurity were found only among households that do not participate in SNAP. In fact, among SNAP participating households, those that were entirely Black had a lower risk of food insecurity than those that were entirely White when accounting for household characteristics. Although SNAP benefits may help address racial economic inequality by providing money for food and despite evidence from prior studies that SNAP participation reduces food insecurity,^3,4,5^ our results contribute to the literature by suggesting that the current SNAP program is not eliminating racial disparities in food insecurity. This situation is likely attributable to the pervasive nature of structural and systemic factors that contribute to racial disparities in food insecurity.^25^

SNAP is the largest US food assistance program,^26^ and SNAP participation reduces the risk of food insecurity in low-income populations by approximately 30%.^3^ This is notable because food insecurity is associated with numerous health conditions, including a higher risk of hyperlipidemia,^27^ diabetes,^28^ functional limitations,^18^ COVID-19,^29^ mortality, and greater health care expenditures.^18^ Importantly, there are well-documented and longstanding racial disparities in these conditions,^30,31,32,33^ suggesting that food insecurity likely contributes to racial health disparities. SNAP participation has also been associated with less health care use,^34,35,36^ less cost-related medication nonadherence,^37^ and, among those with diabetes, better glucose control,^38^ suggesting that participants are better able to manage their health than their peers. Therefore, greater attention should be paid to supporting SNAP as a tool to address racial food insecurity and health disparities.

There are at least 2 different, although likely not mutually exclusive, potential reasons for these study findings. First, it is possible that SNAP benefits are successful in attenuating racial disparities in food insecurity for participants. However, it is also possible that there are race-based differences in access to this critical entitlement program. With regard to the first, there is well-documented structural and systemic racism in financial systems^39,40^ and evidence of racially segregated food environments, including less access to fruits and vegetables^41,42^ and fewer grocery stores^9,10,11,12,13^ in predominately Black communities, which may contribute to higher food costs in those areas.^14,15^ SNAP may partly address these structural barriers to accessing healthy and affordable food. However, the effects of SNAP benefits on food insecurity likely depend partly on the local cost of food,^43^ suggesting that SNAP benefit amounts may need to be better adjusted for cost of living (currently, the rate is higher only for people in Alaska, Hawaii, Guam, and the Virgin Islands). Also, additional interventions and policies are likely needed to address equity in food environments. As an example, a recent review of interventions aiming to improve food security among Black households identified gaps in the development and testing of interventions that address food affordability in communities.^44^ Together, these studies simultaneously highlight the importance of SNAP in combating inequities and identify opportunities to strengthen food assistance programs.

Although SNAP can reduce food insecurity, the results from this study may also be due in part to racial differences in SNAP access. In other words, the findings of differences in food insecurity disparities based on SNAP participation may be because Black households that participate in SNAP are qualitatively different from Black households that do not. Because of pervasive structural racism in the US, it is plausible that there are race-based barriers to SNAP enrollment. The SNAP enrollment process is cumbersome, which has been credited among low-income adults as a reason for nonparticipation,^45^ and there may be requirements that disproportionately affect Black and multiracial households. For example, although SNAP is a federal program, states have flexibility in establishing eligibility criteria and enrollment and recertification requirements, and these state-level differences are associated with state-level participation rates.^46,47^ One study found that changes to the work requirements for working-age adults without dependents were more strongly associated with SNAP participation changes for individuals who were Black vs White, suggesting that these requirements perpetuate structural racism with regard to SNAP access.^48^ Importantly, individuals who are Black are more likely to have a disability^23^ than other racial groups and therefore may experience disproportionate challenges in the SNAP enrollment process because requirements are sometimes inaccessible,^49^ but they are also more likely to live in communities with structural barriers to SNAP enrollment, such as unstable internet access.^50^ Therefore, there is a need to systematically evaluate SNAP program administrative requirements and practices for their potential role in racial disparities in SNAP access.

### Implications

These results have important implications for practice and policy. They highlight the need for universal food insecurity screening, which has been recommended by both the federal government^51^ and the Centers for Medicare & Medicaid Services.^52^ Not only does universal food insecurity screening have potential to reduce racial food insecurity disparities, but timely identification of food insecurity and referral for food assistance has potential to prevent adverse health consequences of food insecurity and thereby reduce racial health disparities.^53^

These findings also have implications for improving SNAP. Despite all study participants being entitled to participate in SNAP and despite its potential benefits, less than 55% of eligible households participated in SNAP across all racial groups, which is comparable with other national estimates.^54^ Findings from this study highlight the need to streamline the SNAP enrollment process.

Importantly, this study’s finding that racial food insecurity disparities are present among those not participating in SNAP suggests that greater attention should be paid to preventing food insecurity. There is an urgent need to address the root causes of racial disparities, including structural and systemic racism in economic systems and food systems. Because food insecurity rates are largely influenced by household and community economic conditions,^18^ addressing the widening racial gaps in income and wealth^40^ should reduce food insecurity disparities. In addition, despite recognition of structural and systemic racism in food systems that shape local food environments, efforts are needed to understand how to address equity across communities.^25^

### Limitations

This cross-sectional study is not intended to draw causal inferences and has limitations. First, SNAP participation may be underreported,^55^ although the SIPP study is designed, in part, to track SNAP participation, and reporting bias would not be expected to differ across racial groups. Second, there may be unmeasured factors that are associated with both SNAP participation and food insecurity. Third, this study examined households with incomes at 130% or less of the poverty threshold based on federal SNAP eligibility limits; therefore, results are not generalizable to the full US population. Fourth, because food insecurity and SNAP participation may influence each other,^3,4,5^ there is possible simultaneity bias in this study.

## Conclusions

This cross-sectional study found racial disparities in food insecurity among low-income adults who do not participate in SNAP but not among those who do participate. Although these results may be due to SNAP’s ability to reduce food insecurity for low-income households, it is also possible that there are systemic barriers to SNAP participation that disproportionately affect minoritized communities. These findings call for greater attention to address structural barriers to affordable food and food assistance that perpetuate racial inequities in food insecurity.

## Notes

Supplement 1. **eTable.** Summary of Survey of Income and Program Participation Variables Used in This Study

Supplement 2.
Data Sharing Statement
