# Supplementary material for: Supplemental Nutrition Assistance Program Access and Racial Disparities in Food Insecurity
Source: JAMA Netw Open. 2023 Jun 26;6(6):e2320196. doi: 10.1001/jamanetworkopen.2023.20196 (PMC10293911; doi:10.1001/jamanetworkopen.2023.20196)
Supplement: Supplement 1. — eTable. Summary of Survey of Income and Program Participation Variables Used in This Study [file jamanetwopen-e2320196-s001.pdf]

Supplementary Online Content

Samuel LJ, Crews DC, Swenor BK, et al. Supplemental Nutrition Assistance Program access and racial disparities in food insecurity. *JAMA Netw Open*. 2023;6(6):e2320196. doi:10.1001/jamanetworkopen.2023.20196

eTable. Summary of Survey of Income and Program Participation Variables Used in This Study

This supplementary material has been provided by the authors to give readers additional information about their work.

© 2023 Samuel LJ et al. *JAMA Network Open.*

eTable. Summary of Survey of Income and Program Participation Variables Used in This Study

Variable	Description
Food insecurity	These next questions are about whether you could afford the food you/your household needed during [year]. I'm going to read you some statements that people have made about their food situations.<br>1. The first statement is, "The food that I/we bought just didn't last, and I/we didn't have money to get more." Was that often, sometimes, or never true for you/your household in [year]?<br>2. I/We couldn't afford to eat balanced meals." Was that often, sometimes, or never true for you/your household in [year]?<br>3. In [year], did you/your household ever cut the size of meals or skip meals because there wasn't enough money for food? (yes/no)<br>4. If yes to #3 → How often did this happen? Was it Almost every month, Some months but not every month, or Only 1 or 2 months<br>5. In [year], did you ever eat less than you felt you should because there wasn't enough money for food? (yes/no)<br>6. In [year], were you ever hungry but didn't eat because there wasn't enough money for food? (yes/no)
SNAP participation	Did you or anyone in your family receipt Food stamps/SNAP benefits since January [year]? And "Just to be sure nothing is missed, at any time since January [year] did you/anyone in your household receive assistance such as Food Stamps/SNAP, WIC, Medicaid, TANF, or General Assistance from a federal, state, or local agency?"

© 2023 Samuel LJ et al. *JAMA Network Open.*

Race	For each person in the household: "Please chose one or more races that you/he/she consider(s) yourself/himself/herself to be?'<br>- White<br>- Black or African American<br>- American Indian or Alaska Native<br>- Asian<br>- Native Hawaiian or Other Pacific Islander<br>- Other [DO NOT READ]
SNAP benefit duration	"When did you/he/she start receiving Food Stamps?" and "When did you/he/she stop?"
SNAP per-person benefit amount	How much do/does you/he/she receive now?
Number of adults in the household	Note: Calculated based on the age of each person on the household roster
U.S. region	Note: Generated by the Census based on the residence for the interview address
Nativity status	Was/were you/he/she born in the United States?
Ethnicity	Is/are you/she/he Spanish, Hispanic or Latino?

© 2023 Samuel LJ et al. *JAMA Network Open.*

Presence of children	Note: Calculated based on the age of each person on the household roster
Presence of older adults	Note: Calculated based on the age of each person on the household roster
Income	Sum of monthly earnings and income received by household members age 15 and older, as well as SSI payments received by children under age 15

© 2023 Samuel LJ et al. *JAMA Network Open.*
