# Supplementary material for: Supplemental Nutrition Assistance Program Access and Racial Disparities in Food Insecurity
Source: JAMA Netw Open. 2023 Jun 26;6(6):e2320196. doi: 10.1001/jamanetworkopen.2023.20196 (PMC10293911; doi:10.1001/jamanetworkopen.2023.20196)
Supplement: Supplement 2. — Data Sharing Statement [file jamanetwopen-e2320196-s002.pdf]

Data Sharing Statement

Samuel. Supplemental Nutrition Assistance Program Access and Racial Disparities in Food Insecurity. *JAMA Netw Open*. Published June 26, 2023.
doi:10.1001/jamanetworkopen.2023.20196

Data
Data available: No

Additional Information
Explanation for why data not available: Data is already publicly available.
